# Supplementary material for: The Construction and Exploration of a Comprehensive MicroRNA Centered Regulatory Network in Foxtail Millet (Setaria italica L.)
Source: Front Plant Sci. 2022 May 6;13:848474. doi: 10.3389/fpls.2022.848474 (PMC9121102; doi:10.3389/fpls.2022.848474)
Supplement: Supplementary file 5 [file Data_Sheet_5.zip › Sit-miR396d_Seita.9G153100.1_1599_TPlot.pdf]

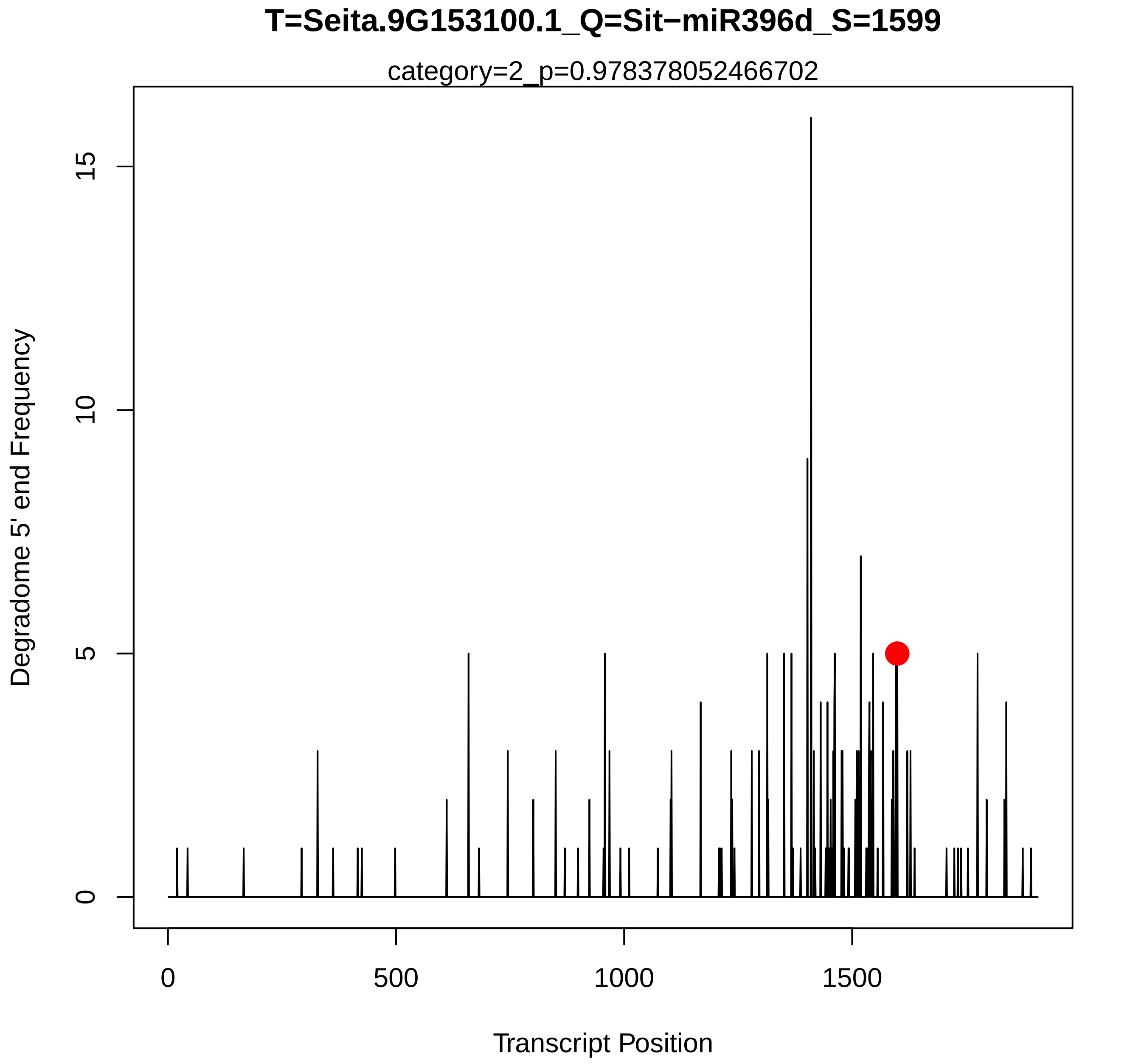

T=Seita.9G153100.1_Q=Sit−miR396d_S=1599
category=2_p=0.978378052466702
Degradome 5' end Frequency
0
5
10
15
0
500
1000
1500
Transcript Position
